# Revisit the Effects of Health Literacy on Health Behaviors in the Context of COVID-19: The Mediation Pathways Based on the Health Belief Model

**DOI:** 10.3389/fpubh.2022.917022

**Published:** 2022-07-13

**Authors:** Huiqiao Zhang, Liyuan Chen, Fan Zhang

**Affiliations:** ^1^Department of Public Health and Preventive Medicine, School of Medicine, Jinan University, Guangzhou, China; ^2^Division of Medical Psychology and Behavior Science, School of Medicine, Jinan University, Guangzhou, China

**Keywords:** healthy literacy, the health belief model, health behaviors, COVID-19, mediation effect

## Abstract

**Background:**

Emerging research has identified health literacy as an important resource for individual health care and disease prevention. In the context of COVID-19, People with limited HL are less likely to follow preventive measures such as wearing masks, social isolation, or taking the vaccination. However, the pathways of how health literacy affects decision-making have remained unclear.

**Methods:**

With a cross-sectional study, a total of 613 responses (mean age is 25.64 ± 6.46 years) were collected. The relationship between health literacy and health behaviors under COVID-19 was examined, and the potential mediation pathways were assessed based on the health belief model.

**Results:**

With linear regression, it was found that health literacy has a direct effect on health behaviors and three constructs in the health belief model, i.e., perceived susceptibility, perceived severity, perceived barriers, as well as an indirect effect on health behaviors via increasing perceived barriers related with COVID-19 preventive measures. The results showed that health literacy only goes through the pathway of perceived barriers to influence health behaviors, and the indirect effects via other pathways were not significant.

**Conclusions:**

The research addressed the mediation model underlying the effects of health literacy on health behaviors and identified a partial mediation role of perceived barriers. Health literacy could promote individual health behavior by reducing the perceived barriers to forming a healthy lifestyle and making health decisions. Future health promotion interventions increasing people's health literacy should be advocated to promote health initiatives in the whole population.

## Introduction

From the outbreak of COVID-19 in early 2020 (1) till May 9, 2022, more than 500 million confirmed cases infected with COVID-19 were reported, and over six million deaths (2). However, no effective treatment has been developed yet, and only preventive measures such as vaccination could be adopted to control the spread of the virus. During conducting this study (July to August 2021), China had a large-scale COVID-19 vaccination campaign, with 43.2% of the population having received at least one shot of COVID-19 vaccination (3). At the same time, other preventive measures were promoted including crowd avoidance (4, 5), handwashing, and wearing masks, which are considered essential means to fight against COVID-19 by WHO (6). On the other hand, personal healthy lifestyles have received increased awareness in healthcare and disease prevention, such as regular medical checkups (7), frequent exercises to improve people's physical health and immunity (8). However, not everyone can follow the instructions to implement these preventive measures.

Health literacy (HL) was defined as “the ability to identify, understand, evaluate and use the information to improve decision-making and ultimately health and quality of life” (9). Health literacy was found to be an important factor that motives people to follow preventive measures and adopt healthy lifestyles (10, 11). Previous study has found that people with sufficient HL are more likely to have preventive behaviors (12). According to the framework of HL, vaccine literacy (also known as “vaccine-related HL”) refers to “a process of providing vaccine information, building communication, and increasing people's engagement about vaccines” (13), and both HL and vaccine literacy have obtained increasing research attention in during the pandemic. Previous studies have shown that limited HL is associated with lower engagement in a variety of preventive health behaviors (14, 15), including mammograms (16), influenza immunizations (17), and weight management (18). In particular, lacking the ability to understand health information could lead to deliberately ignoring recommended preventive measures, thus increasing the risk of infection and mortality (19, 20). Interventions were developed to increase people's HL, however, without a better understanding of the underlying mechanism, increased HL may not be effectively transformed into people's healthy life.

However, how HL works in affecting health behaviors has remained a black box. Theoretical models such as the health belief model (HBM) were developed to identify the factors that influence behavioral change in healthcare and disease prevention (21).

According to HBM (22, 23), people's behavioral changes depend on their perceived risk of infection and severity of the disease (perceived susceptibility and perceived severity), as well as the perceived benefits and barriers in the behavioral change (perceived benefits and perceived barriers). According to the Health Belief Model (24) and the HL Skills Framework (25), it was proposed that HL may influence behavioral outcomes via shaping people's beliefs about the health motivation of the outcomes (26) Previous studies have supported the HBM as a useful model for predicting/explaining preventive behavior toward infectious diseases such as COVID-19 (26, 27). Niu et al. (28) have found that HBM constructs were both associated with lower engagement in preventive behaviors among high-risk Chinese. According to this model, the likelihood of a person participating in a healthy activity is based on personal beliefs. For example, people will adopt health behaviors when they feel threatened by an epidemic (perceived susceptibility) or believe that a disease may have a serious impact on their health (perceived severity). Meanwhile, HL will affect how useful they perceive the preventive measures, such as using masks (perceived benefits), and how difficult they perceive the particular behaviors (perceived barriers) (26, 29). By increasing perceived benefits, perceived sensitivity, perceived intensity, and self-efficacy and raising awareness, HL can reduce barriers to engaging in health behaviors (30, 31). Brega et al. (32) found that stronger HL was associated with more positive health beliefs (i.e., perceived severity and benefits, and lower perceived barriers). Therefore, it is possible that HL may influence health behaviors by shaping people's health beliefs.

**Figure 1:**
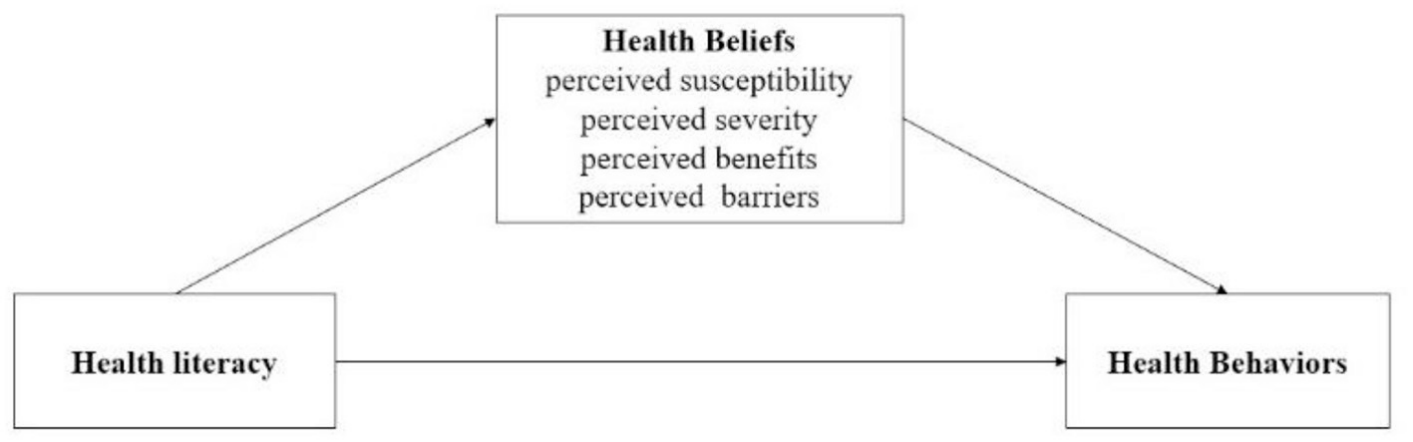
Multi-mediation model.

Considering the importance of health behaviors in controlling and preventing the spread of COVID-19, the current study has considered health behaviors including both lifestyle behaviors and COVID-19 related preventive behaviors, such as smoking, drinking, wearing masks, and so on. Despite previous studies have examined the effects of health literacy and health beliefs on health behavior, few has focused on the mechanisms underlying the relationship between health literacy on health behaviors. Therefore, to address this issue, our study has integrated the health belief model and health literacy skills framework, and investigated the effect and mediation mechanism of HL on health behaviors during the COVID-19 pandemic. We have therefore developed a multi-mediation model (see Figure 1), which proposed that HL influenced health behaviors both directly and indirectly via the pathways of different health beliefs. The model outlines the following hypotheses: (1) HL may influence people's health beliefs about COVID-19 (perceived susceptibility and severity) and perception about the preventive measures (e.g., taking COVID-19 vaccine) against COVID-19 (perceived benefits and barriers); (2) People's health beliefs may explain (at least partially) the relationship between HL and health behaviors.

## Methods

### Study Design

With a cross-sectional design, the data was collected from July to August, 2021 via an online questionnaire platform “Wenjuanxing” (Ranxing Information Technology Co., Ltd., Changsha, Hunan, China). It is similar to Qualtrics, SurveyMonkey, or CloudResearch, which has been widely recognized by top journals in social science. Participants were recruited from community clinics, and inclusion criteria were: (1) having the ability and access to online devices; (2) being native Chinese speakers. With a convenience sampling method, the survey was administered with face-to-face interview at Health Screening Centers and community clinics in Guangzhou. After obtaining the written consent from the participants, they would receive a link of the survey on their mobile phone. The survey was voluntary and anonymous for participants, and each IP address can only access the link once to eliminate repeated submission. No personal information or sensitive content was included in the questionnaire. Only the participants who provided informed consent would take part in the online survey. The study was approved by the Jinan University Human Research Ethic Committee.

### Data Collection

In total, 662 participants have completed the questionnaire. Based on our previous studies using online survey [e.g., (33)], those with consecutively repeated answers were excluded. Afterwards, 613 valid responses (92.60%) were included in the analysis.

### Measures

#### Demographic Information

Demographic variables, including age, gender, marital status, education level, chronic conditions, and health behaviors were asked. And some of these are treated as covariates: age, gender (0 = male, 1 = female), marital status (0 = married, 1 = single/separated/divorced/widowed/others), education level (1 = primary school, 2 = junior high school, 3 = senior middle school, 4 = university and above), and chronic conditions (0 = yes, 1 = no).

#### HL

HL was measured using a short version of the European Health Literacy Questionnaire (HLS-EU-Q) (i.e., HLS-SF12) (34), consisting of 12 items assessing information processing skills in the areas of health promotion, health care, and disease prevention, to which we added three vaccine-related items according to the study design (e.g., “I can understand why I need to be vaccinated”). A 4-point Likert scale was used, with responses ranging from “1”(very difficult) to “4”(very easy), and “don't know” was coded as a missing value. The mean score indicates the level of HL of the individual, with higher scores indicating better HL (35). The Cronbach's alpha coefficient for our sample was 0.934, indicating good internal consistency.

#### HBM Constructs

The HBM constructs was based on the research by Shahrabani and Benzion (21). The questionnaire included the following questions on the structure of the HBM: HBM_1_-Perceived susceptibility (three items); HBM_2_-Perceived severity (three items); HBM_3_- Perceived benefit (three items); HBM_4_- Perceived barrier (four items); All items were scored using a five-point Likert scale ranging from strongly agree “1” to strongly disagree “5” (see Table 1). Scores on each scale were averaged to generate the mean score, with higher mean scores indicating lower levels of agreement with their corresponding constructs. The structures of the HBM categories have been tested for reliability and validity in previous studies (36). The Cronbach's alpha coefficient for our sample was 0.796.

**Table 1:** Health belief model questionnaires.

**Variables**	**Statements^**#**^**
Susceptibility	Working with many people each day increases my chances of getting the COVID-19
	My chances of getting the COVID-19 are good
	I worry a lot about getting the COVID-19
Severity	Getting the COVID-19 would disrupt my family
	Having the COVID-19 would make daily activities more difficult
	COVID-19 can be a serious disease
Benefits	Getting a COVID-19 shot will prevent me from getting the COVID-19
	Getting a COVID-19 shot will prevent me from missing work
	I would not be afraid of getting the COVID-19 if I got a COVID-19 shot
Barriers	Getting a COVID-19 shot can be painful
	Getting a COVID-19 shot is time consuming
	There are too many risks in getting a COVID-19 shot
	I am concerned about having a bad reaction to the COVID-19 shot

^#^*The options for the items ranged from strongly agree (1) to strongly disagree (5)*.

#### Health Behaviors

Health behaviors were selected based on the recommendations of the World Health Organization (6). Both lifestyle behaviors (e.g., exercise, alcohol consumption, medical check-ups, smoking) and preventive behaviors related to COVID-19 (e.g., wearing masks, washing hands, crowd avoidance, taking COVID-19 and influenza vaccination) were assessed. For example, in terms of lifestyle behaviors, participants were asked if they regularly take part in the exercise, drink alcohol, smoke, or take medical check-ups with the options of “yes” or “no.” We also asked participants if they had received any influenza vaccine in the past year. Regarding preventive behaviors, participants were asked how they have avoided crowded places, wore masks, and washed their hands in the last 2 weeks with the options from “never”; “occasionally”; “often”; “always.” We also asked participants if they had received the COVID-19 vaccine with the options of (received “one shot”; “two shots”; or “no”). To generate a total score of various health behaviors, the rating of each item was transformed into a binary variable (0: “no or infrequent,” 1: “yes or frequent”), and the sum was obtained by counting the total number “1.” With a score ranging from 0 to 9, higher scores indicate more health behaviors.

### Data Analysis

Multiple regression analyses were conducted to examine the associations between health beliefs, HL, demographic variables, and health behaviors. The PROCESS macro was used to test the mediating relationships proposed in the hypothesis (37). The PROCESS model 4 was used to examine mediation pathways. All data were analyzed using the Statistical Package for the Social Sciences (SPSS) version 20. Age, gender, marital status, education, and chronic illness were controlled as covariates in multiple regression models and mediation analyses. Alpha error probability <0.05 was considered statistically significant (two-sided test).

## Results

### Descriptive Results

Table 2 summarizes the demographic information and characteristics of the sample. A sample of 613 participants was included in the analysis. The mean age of the sample was 25.64 years (SD = 6.46), ranging from 14 to 60 years old, and 53.3% were male. The majority of the participants were not married (79.1%), obtained a university degree or above (65.7%), and had no chronic conditions (97.6%). The average number of health behaviors was 6.24 (SD = 1.16), suggesting that in general people have adopted most health behaviors. The mean HL score was 3.37 (SD = 0.44), indicating that most people responded between “easy” and “very easy”. For the Health Beliefs Model, HBM_1_-Susceptibility had a mean score of 3.28 (SD = 0.78), HBM_2_- Severity had a mean score of 2.18 (SD = 0.92), HBM_3_-Benefits had a mean score of 2.70 (SD = 0.79), and HBM_4_-Barriers had a mean score of 3.59 (SD = 0.73). Despite that, the majority of our sample reported not using tobacco or alcohol (“no smoking”: 78.0%; “no drinking”: 91.5%), the descriptive analysis showed that during the pandemic, only 34.7% of participants reported doing regular exercise, 48.3% had regular medical check-ups and only 8.8% received a flu vaccination in the past year. Regarding preventive behaviors related to the pandemic, over 80% of the participants reported wearing the mask, avoiding crowds, and having taken at least one shot of the COVID-19 vaccine.

**Table 2:** The descriptive results of the participants (*N* = 613).

	***N* (% of sample)** **Mean ±S.D**.	**Range**
**Gender**		
Male (0)	327 (53.3%)	
Female (1)	286 (46.7%)	
**Age**	25.64 ± 6.46	14–60
**Marital status**		
Married (0)	128 (20.9%)	
Single/Separated/Divorced/ Widowed/Others (1)	484 (79.1%)	
**Education level**		
Primary school (1)	2 (0.3%)	
Junior high school (2)	43 (7.0%)	
Senior middle school (3)	165 (26.9%)	
University and above (4)	403 (65.7%)	
**Chronic conditions**		
Yes (0)	15 (2.4%)	
No (1)	597 (97.6%)	
Health behaviors	6.24 ± 1.16	0–9
Health literacy	3.37 ± 0.44	1,4
**Health beliefs**		
HBM_1_-Susceptibility	3.28 ± 0.78	1,5
HBM_2_- Severity	2.18 ± 0.92	1,5
HBM_3_-Benefits	2.70 ± 0.79	1,5
HBM_4_-Barriers	3.59 ± 0.73	1,5

*HBM, the Health Belief Model*.

### The Effects of HL

Table 3 shows the correlation between HL, health behaviors and health beliefs. The linear regression model was conducted to examine the effects of HL on different dependent variables one by one, with controlling for socio-demographic characteristics such as age, gender, and education level (see Table 4). It was found that higher education was associated with more health behaviors (β = 0.214, *p* < 0.001). After controlling for the covariates, greater HL was also associated with more health behaviors (β = 0.319, *p* < 0.001). HL was also associated with less perceived susceptibility (β = 0.267, *p* < 0.001), perceived severity (β = 0.170, *p* = 0.043) of COVID-19, and fewer perceived barriers (β = 0.371, *p* < 0.001), but no effect on perceived benefit was found.

**Table 3:** The correlation matrix between health behaviors, health literacy and health beliefs.

	**1**	**2**	**3**	**4**	**5**	**6**	**7**	**8**	**9**	**10**
1.Age	1									
2.Gender	−0.196**	1								
3.Marital status	−0.623**	0.135**	1							
4.Educational level	−0.184**	0.344**	0.255**	1						
5.Chronic illness	−0.070	−0.085*	0.023	−0.088*	1					
6.Health literacy	−0.094*	−0.001	0.004	0.000	−0.026	1				
7.HBM_1_-Susceptibility	−0.063	−0.054	0.090*	−0.066	−0.038	0.152**	1			
8.HBM_2_- Severity	−0.047	−0.093*	0.027	−0.067	−0.031	0.088*	0.431**	1		
9.HBM_3_-Benefits	−0.082*	0.008	0.083*	0.088*	−0.087*	−0.040	0.134**	0.361**	1	
10.HBM_4_-Barriers	−0.099*	−0.072	0.116**	−0.022	0.037	0.223**	0.374**	0.143**	0.135**	1
11.Health behaviors	0.089*	0.014	−0.094*	0.076	0.024	0.110**	0.042	0.071	−0.048	0.119**

**p < 0.01. *p < 0.05.

**Table 4:** Linear regression predicting health beliefs and health behaviors.

	**Dependent variable** ^**Δ**^				
**Predictors**	**HBM_**1**_ - susceptibility,** **coefficient (SE)**	**HBM_**2**_ - severity,** **coefficient (SE)**	**HBM_**3**_ - benefits,** **coefficient (SE)**	**HBM_**4**_ - barriers,** **coefficient (SE)**	**Health behaviors,** **coefficient (SE)**
Age	0.002 (0.006)	−0.005 (0.007)	−0.005 (0.006)	0.000 (0.006)	0.015 (0.010)
Gender	−0.050 (0.067)	−0.144 (0.079)	−0.042 (0.067)	−0.091 (0.062)	0.010 (0.101)
Marital status	0.229* (0.100)	0.050 (0.117)	0.056 (0.100)	0.209* (0.093)	−0.212 (0.150)
Educational level	−0.081 (0.053)	−0.039 (0.063)	0.125* (0.053)	−0.015 (0.050)	0.214** (0.080)
Chronic illness	−0.271 (0.207)	−0.290 (0.244)	−0.391 (0.207)	0.149 (0.193)	0.332 (0.312)
Health literacy	0.267** (0.071)	0.170* (0.084)	−0.083 (0.071)	0.371** (0.066)	0.319** (0.107)
*R* ^2^	0.039	0.018	0.025	0.068	0.038

**p < 0.01. *p < 0.05. Coefficient is unstandardized. SE, Standard errors.

^Δ^*The results were controlled for age, gender, marital status, education level, and chronic conditions*.

### The Mediating of HBM in the Effect of HL

**Figure 2:**
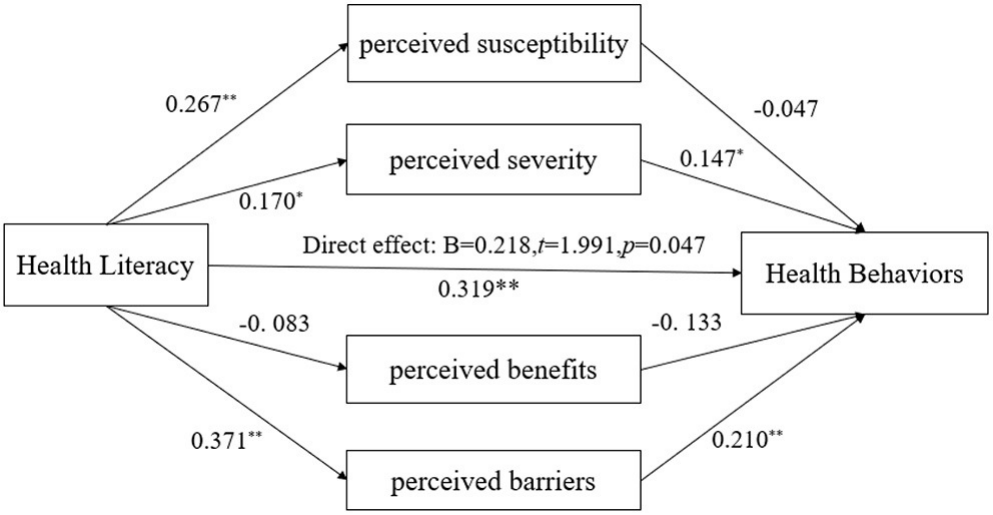
A mediation model of health beliefs between health literacy and health behaviors. The model has adjusted for age, gender, marital status, chronic conditions, and education levels. *p < 0.05, **p < 0.01.

As abovementioned, after adjusting for age, gender, educational level, marital status, and whether has a chronic illness, a higher level of HL was associated with more health behaviors (β = 0.319, *p* < 0.001, see Table 4). In addition, lower perceived severity and perceived barriers were also found to be related to more health behaviors (HBM_2_- Severity: β = 0.147, *p* < 0.05; HBM_4_-Barriers: β = 0.210, *p* < 0.001). In particular, mediation analysis showed that the effects of HL were partially mediated via reduced perceived barriers (B = 0.078, 95%CI = [0.020, 0.146]), but not via perceived susceptibility (B = −0.013, 95%CI = [−0.051,0.026]), severity (B =0.025, 95%CI = [−0.000,0.065]), or benefits (B = 0.011, 95%CI = [−0.008, 0.043]) Note that this mediation effect was only partial, and after including the HBM constructs in the regression model, the direct effect of HL on increasing health behaviors has remained significant (B = 0.218, *p* = 0.047), see Figure 2.

## Discussion

The current study investigated how HL affected health behaviors based on the health belief model. HL was found to be associated with a lower level of perceived susceptibility, perceived severity, and perceived barriers, while it was related to more health behaviors. In addition, the positive effect of HL was partially explained by reducing perceived barriers to adopting preventive behaviors with COVID-19. The results may provide insight for developing interventions for promoting the adoption of health behaviors in the context of the COVID-19 pandemic.

Despite the average number of health behaviors being about six in our sample, when looking at specific behaviors, the prevalence has remained low. Recent evidence showed that people spent less time on physical exercise during the pandemic. For example, 56% of respondents in an Italian sample reported spending less time on physical activity, particularly among older adults and residents of large cities (38); while only 4.8% of children and 0.6% of adolescents in Canada followed exercise guidelines standards (39); and inconsistent, sedentary leisure behavior increased among US adults, while physical activity time has declined (39). Across the studies in different samples, it was shown that the frequency of physical exercise has declined in different populations. As for preventive behaviors, the prevalence is also unsatisfactory. When asked whether they had received an influenza vaccination in the past year, only 8.8% of our participants reported doing so. Although there is a higher than that was found in the 2014–15 influenza vaccination survey in China (2.4%) (40), but has remained low. In comparison, during the 2018–2019 influenza season in the United States, influenza vaccination rates were about 49% (41). Low rate of engaging in physical exercise and taking the influenza vaccine could contribute to greater morbidity and mortality during the pandemic of COVID-19 (41, 42). Therefore, it is urgent to develop interventions that could effectively promote people's health behaviors during the pandemic to mitigate the negative impact caused by COVID-19.

People with higher level of HL were more likely to form healthy lifestyles and adopt preventive measures. Many studies on the relationship between HL and health behaviors have supported the protective effects of HL (20, 43–46), prompting us to consider improving HL as a critical measure to curb the pandemic (47). Better understanding, appreciation, and application of health information can support actions at multi-level to address major public health challenges (48). As far as our knowledge, little research has explored the potential mechanism underlying HL on behaviors. Adopting the theoretical framework of the health belief model, we found that HL was significantly associated with lower perceived susceptibility, perceived severity, and perceived barriers. Moreover, lower perceived severity or barriers were associated with more health behaviors, while the causal relationship is not clear. e.g., it is also possible that people who adopt healthcare or preventive measures may develop better sense of security and perceive lower level of severity. Longitudinal studies are needed to explore the causal relationships.

In addition, the findings provide further evidence on the underlying mechanism of HL via influencing different health beliefs. Previous research has confirmed the role of HL on the structures of HBM (49, 50), and this framework provides a useful tool to modify and explain the effects of HL (50). Previous study has found that greater perceived barriers and less perceived benefits were both associated with lower engagement in preventive behaviors among high-risk Chinese, and perceived barriers and benefits, self-efficacy, and trust in media and doctors have fully mediated the effects of HL in predicting preventive behaviors (28). In our study, however, the perceived benefit was not found to effectively predict health behaviors, possibly because our sample was recruited from a more general population of which the majority was free from chronic conditions and perceived lower benefits of health behaviors, thus muting the potential effects. Consistent with the theoretical framework of Squiers et al. (25), we found that perceived barriers was an important predictor of health behaviors. Meanwhile, greater HL was associated with reduced perceived barriers, which may be because people with sufficient HL could apply various health information and handle different healthcare needs more appropriately (51), thus easily solving various obstacles. It is also possible that HL is related to greater initiative in healthcare and disease prevention (52), which motivates people to overcome the barriers. Besides, HL is closely related to health empowerment (53), indicating a higher level of meaningfulness, competence, impact, and self-determination in managing health-related situations (54), which could also contribute to lower perceived barriers.

There are some limitations to our study. First, with a cross-sectional survey, it is difficult to address the causal relationships, and longitudinal or intervention studies should be conducted. Second, we used the convenience sampling method, which may lead to selection bias and challenge the generalizability of the conclusions to other samples. Future studies could be conducted on health behaviors across different populations.

## Conclusion

With the theoretical framework of HBM, the current study has investigated the mechanism of HL in promoting health behavior in the context of COVID-19. Results showed that in addition to a direct effect on increasing health behaviors, HL also had an indirect effect via reducing people's perceived barriers to adopting these behavioral measures. Our findings support the idea that HL plays a critical role in the adoption of health behaviors during the pandemic. This finding also provides a theoretical basis for governments, public health agencies, and healthcare professionals to develop effective policy and interventions promoting people's initiative and self-discipline in forming a healthy lifestyle, to better adapt to the post-pandemic era.

## Data Availability Statement

The raw data supporting the conclusions of this article is available from the corresponding author on request.

## Ethics Statement

The studies involving human participants were reviewed and approved by the Ethics Committee of Jinan University. Written informed consent from the participants' legal guardian/next of kin was not required to participate in this study in accordance with the national legislation and the institutional requirements.

## Author Contributions

FZ and HZ has conceived of the presented idea, analyzed the data, and written the manuscript. FZ, HZ, and LC has designed the survey. HZ has performed data collected. All authors reviewed the final version. All authors contributed to the article and approved the submitted version.

## Funding

The study was supported by the Humanity and Social Science Youth foundation (21YJCZH209), MoE.

## Conflict of Interest

The authors declare that the research was conducted in the absence of any commercial or financial relationships that could be construed as a potential conflict of interest.

## Publisher's Note

All claims expressed in this article are solely those of the authors and do not necessarily represent those of their affiliated organizations, or those of the publisher, the editors and the reviewers. Any product that may be evaluated in this article, or claim that may be made by its manufacturer, is not guaranteed or endorsed by the publisher.

## Abbreviations

HBM: the Health Belief Model
COVID-19: Coronavirus disease 2019
HL: Health literacy
SPSS: the Statistical Package for the Social Sciences.

## References

[1] Emanuel EJ Persad G Upshur R Thome B Parker M Glickman A. Fair allocation of scarce medical resources in the time of Covid-19. N Engl J Med. (2020) 382:2049–55. 10.1056/NEJMsb2005114 32202722

[2] WHO. Who Coronavirus (Covid-19) Dashboard. (2022). Available online at: https://covid19.who.int/ (accessed May 9, 2022)

[3] Erika Fry, Rapp. N. 25.6% of the World Has Received a Covid Vaccine. See How Your Country Is Doing: FORTUNE (2021). Available online at https://fortune.com/2021/07/14/covid-vaccine-tracker-update-worldwide-us-countries-pfizer-moderna-johnson-johnson-numbers-data/

[4] Wang C Chudzicka-Czupała A Grabowski D Pan R Adamus K Wan X. The association between physical and mental health and face mask use during the Covid-19 pandemic: a comparison of two countries with different views and practices. Front Psychiatry. (2020) 11:569981. 10.3389/fpsyt.2020.569981 33033485 PMC7510452

[5] Chen S Yang J Yang W Wang C Bärnighausen T. Covid-19 Control in China during mass population movements at new year. Lancet). (2020) 395:764–6. 10.1016/S0140-6736(20)30421-9 32105609 PMC7159085

[6] WHO. Advice for the Public: Coronavirus Disease (Covid-19) (2021) [1 October]. Available online at: https://www.who.int/emergencies/diseases/novel-coronavirus-2019/advice-for-public

[7] Tan W Hao F McIntyre RS Jiang L Jiang X Zhang L. Is Returning to Work During the Covid-19 Pandemic Stressful? A study on immediate mental health status and psychoneuroimmunity. Prevent Measu Chinese Workforce Brain Behav Immun. (2020) 87:84–92. 10.1016/j.bbi.2020.04.055 32335200 PMC7179503

[8] Nyenhuis SM Greiwe J Zeiger JS Nanda A Cooke A. Exercise and fitness in the age of social distancing during the Covid-19 pandemic. J Allergy Clin Immunol Pract. (2020) 8:2152–5. 10.1016/j.jaip.2020.04.039 32360185 PMC7187829

[9] Sørensen K Pleasant A. Understanding the conceptual importance of the differences among health literacy definitions. Stud Health Technol Inform. (2017) 240:3–14. 10.3233/978-1-61499-790-0-3 28972505

[10] Lastrucci V Lorini C Del Riccio M Gori E Chiesi F Moscadelli A. The role of health literacy in Covid-19 preventive behaviors and infection risk perception: evidence from a population-based sample of essential frontline workers during the lockdown in the province of prato (Tuscany, Italy). Int J Environ Res Public Health. (2021) 18:3386. 10.3390/ijerph182413386 34948995 PMC8702135

[11] Do BN Tran TV Phan DT Nguyen HC Nguyen TTP Nguyen HC. Health literacy, ehealth literacy, adherence to infection prevention and control procedures, lifestyle changes, and suspected Covid-19 symptoms among health care workers during lockdown: online survey. J Med Internet Res. (2020) 22:e22894. 10.2196/22894 33122164 PMC7674138

[12] Nutbeam D. Health literacy as a public health goal: a challenge for contemporary health education and communication strategies into the 21st century. Health Promot Int. (2000) 15:259–67. 10.1093/heapro/15.3.259

[13] Badua AR Caraquel KJ Cruz M Narvaez RA. Vaccine literacy: a concept analysis. Int J Ment Health Nurs. (2022). 10.1111/inm.12988 35289065 PMC9111838

[14] Berkman ND Sheridan SL Donahue KE Halpern DJ Crotty K. Low health literacy and health outcomes: an updated systematic review. Ann Intern Med. (2011) 155:97–107. 10.7326/0003-4819-155-2-201107190-00005 21768583

[15] Sun X Shi Y Zeng Q Wang Y Du W Wei N. Determinants of health literacy and health behavior regarding infectious respiratory diseases: a pathway model. BMC Public Health. (2013) 13:261. 10.1186/1471-2458-13-261 23521806 PMC3621712

[16] Bennett IM Chen J Soroui JS White S. The contribution of health literacy to disparities in self-rated health status and preventive health behaviors in older adults. Ann Fam Med. (2009) 7:204–11. 10.1370/afm.940 19433837 PMC2682976

[17] Sudore RL Mehta KM Simonsick EM Harris TB Newman AB Satterfield S. Limited literacy in older people and disparities in health and healthcare access. J Am Geriatr Soc. (2006) 54:770–6. 10.1111/j.1532-5415.2006.00691.x 16696742

[18] Sharif I Blank AE. Relationship between child health literacy and body mass index in overweight children. Patient Educ Couns. (2010) 79:43–8. 10.1016/j.pec.2009.07.035 19716255 PMC2839034

[19] Abdel-Latif MMM. The Enigma of health literacy and Covid-19 pandemic. Public Health. (2020) 185:95–6. 10.1016/j.puhe.2020.06.030 32593054 PMC7303647

[20] Matterne U Egger N Tempes J Tischer C Lander J Dierks ML. Health literacy in the general population in the context of epidemic or pandemic coronavirus outbreak situations: rapid scoping review. Patient Educ Couns. (2021) 104:223–34. 10.1016/j.pec.2020.10.012 33109429 PMC7547635

[21] Shahrabani S Benzion U. How experience shapes health beliefs: the case of influenza vaccination. Health Edu Behav: Off Publ Soc Public Health Edu. (2012) 39:612–9. 10.1177/1090198111427411 22287574

[22] Pang Q Meng H Fang M Xing J Yao J. Social distancing, health concerns, and digitally empowered consumption behavior under Covid-19: a study on livestream shopping technology. Front Public Health. (2021) 9:748048. 10.3389/fpubh.2021.748048 34604167 PMC8481574

[23] Yao J Pang Q Zhang B Wang L Huang Y. Public health and online mice technology during the Covid-19 pandemic: the role of health beliefs and technology innovation. Front Public Health. (2021) 9:756987. 10.3389/fpubh.2021.756987 34660525 PMC8517434

[24] Champion VL Skinner CS. The Health Belief Model. San Francisco, CA: Jossey-Bass (2008). p. 45–65.

[25] Squiers L Peinado S Berkman N Boudewyns V McCormack L. The health literacy skills framework. J Health Commun. (2012) 17 Suppl 3:30–54. 10.1080/10810730.2012.713442 23030560

[26] Tadesse T Alemu T Amogne G Endazenaw G Mamo E. Predictors of coronavirus disease 2019 (Covid-19) prevention practices using health belief model among employees in Addis Ababa, Ethiopia, 2020. Infect Drug Resist. (2020) 13:3751–61. 10.2147/IDR.S275933 33122922 PMC7588498

[27] Shewasinad Yehualashet S Asefa KK Mekonnen AG Gemeda BN Shiferaw WS Aynalem YA. Predictors of adherence to Covid-19 prevention measure among communities in North Shoa Zone, Ethiopia based on health belief model: a cross-sectional study. PLoS ONE. (2021) 16:e0246006. 10.1371/journal.pone.0246006 33481962 PMC7822535

[28] Niu Z Qin Z Hu P Wang T. Health beliefs, trust in media sources, health literacy, and preventive behaviors among high-risk Chinese for Covid-19. Health Comm. (2021) 10:1–9. 10.1080/10410236.2021.1880684 33557620

[29] Walrave M Waeterloos C Ponnet K. Adoption of a contact tracing app for containing Covid-19: a health belief model approach. JMIR Public Health Surveill. (2020) 6:e20572. 10.2196/20572 32755882 PMC7470174

[30] Panahi R Ebrahimi G Kazemi S Tavousi M. Health Literacy: An effective component to overcome perceived barriers to adoption of preventive behaviors in the health belief model. J Edu Comm Health. (2018) 5:1–3. 10.21859/jech.5.3.1

[31] Aval M Ansari-Moghadam A Masoudy G. Educational intervention based on health belief model on the adoption of preventive behaviors of Crimean-Congo hemorrhagic fever in ranchers. Health Scope. (2019) 8:e14112. 10.5812/jhealthscope.14112

[32] Brega AG Johnson RL Schmiege SJ Wilson AR Jiang L Albino J. Pathways through which health literacy is linked to parental oral health behavior in an American Indian tribe. Annals Behav Med: Pub Soc Behav Med. (2021) 55:1144–55. 10.1093/abm/kaab006 33830175 PMC8557384

[33] Ying Y Zhang F Jing C. The protective effect of health literacy on reducing college students' stress and anxiety during the Covid-19 pandemic. Front Psych. (2022) 4:885. 10.3389/fpsyt.2022.878884 35664470 PMC9161275

[34] Duong TV Aringazina A Kayupova G Nurjanah N Pham TV Pham KM. Development and validation of a new short-form health literacy instrument (Hls-Sf12) for the general public in six Asian countries. HLRP: Health Literacy Res Pract. (2019) 3:e91–e102. 10.3928/24748307-20190225-012022/04/27 31294310 PMC6607763

[35] van der Heide I Rademakers J Schipper M Droomers M Sørensen K Uiters E. Health literacy of dutch adults: a cross sectional survey. BMC Public Health. (2013) 13:179. 10.1186/1471-2458-13-179 23445541 PMC3599856

[36] Shahrabani S Benzion U. Workplace vaccination and other factors impacting influenza vaccination decision among employees in Israel. Int J Environ Res Public Health. (2010) 7:853–69. 10.3390/ijerph7030853 20617008 PMC2872324

[37] Hayes AF Rockwood NJ. Regression-based statistical mediation and moderation analysis in clinical research: Observations, recommendations, and implementation. Behav Res Ther. (2017) 98:39–57. 10.1016/j.brat.2016.11.001 27865431

[38] Ferrante G Camussi E Piccinelli C Senore C Armaroli P Ortale A. Did social isolation during the SARS-CoV-2 epidemic have an impact on the lifestyles of citizens? Epidemiologia e prevenzione. (2020) 44(5-6 Suppl 2):353–62. 10.19191/ep20.5-6.S2.137 33412829

[39] Moore SA Faulkner G Rhodes RE Brussoni M Chulak-Bozzer T Ferguson LJ. Impact of the Covid-19 virus outbreak on movement and play behaviours of Canadian children and youth: a national survey. Int J Behav Nutr Phys Act. (2020) 17:85. 10.1186/s12966-020-00987-8 32631350 PMC7336091

[40] Fan J Cong S Wang N Bao H Wang B Feng Y. Influenza vaccination rate and its association with chronic diseases in China: results of a national cross-sectional study. Vaccine. (2020) 38:2503–11. 10.1016/j.vaccine.2020.01.093 32046892

[41] Conlon A Ashur C Washer L Eagle KA Hofmann Bowman MA. Impact of the influenza vaccine on Covid-19 infection rates and severity. Am J Infect Control. (2021) 49:694–700. 10.1016/j.ajic.2021.02.012 33631305 PMC7899024

[42] Vancini RL Andrade MS Viana RB Nikolaidis PT Knechtle B Campanharo CRV. Physical exercise and Covid-19 pandemic in pubmed: two months of dynamics and 1 year of original scientific production. Sports medicine and health science. (2021) 3:80–92. 10.1016/j.smhs.2021.04.004 34189482 PMC8105136

[43] Nguyen HT Do BN Pham KM Kim GB Dam HTB Nguyen TT. Fear of Covid-19 scale-associations of its scores with health literacy and health-related behaviors among medical students. Int J Environ Res and public health. (2020) 17:4164. 10.3390/ijerph17114164 32545240 PMC7311979

[44] Patil U Kostareva U Hadley M Manganello JA Okan O Dadaczynski K. Health literacy, digital health literacy, and Covid-19 pandemic attitudes and behaviors in .S. college students: implications for interventions. Int J Environ Res Public Health. (2021) 18:3301. 10.3390/ijerph18063301 33806763 PMC8004744

[45] Nakayama K Yonekura Y Danya H Hagiwara K. Covid-19 Preventive behaviors and health literacy, information evaluation, and decision-making skills in Japanese adults: cross-sectional survey study. JMIR formative research. (2022) 6:e34966. 10.2196/34966 34982036 PMC8822428

[46] Yusefi AR Barfar E Daneshi S Bayati M Mehralian G Bastani P. Health literacy and health promoting behaviors among inpatient women during Covid-19 pandemic. BMC Women's Health. (2022) 22:77. 10.1186/s12905-022-01652-x 35300684 PMC8929241

[47] Paakkari L Okan O. Covid-19: health literacy is an underestimated problem. The Lancet Public health. (2020) 5:e249–e50. 10.1016/S2468-2667(20)30086-4 32302535 PMC7156243

[48] Sentell T Vamos S Okan O. Interdisciplinary perspectives on health literacy research around the world: more important than ever in a time of Covid-19. Int J Environ Res Public Health. (2020) 17(9). Epub 2020/05/03. 10.3390/ijerph17093010 32357457 PMC7246523

[49] Rahman P Ali R Mahmoud T Aliasghar H Niknami S. Reinforcing the performance of health belief model using health literacy in anticipating adoption of smoking preventive behaviors in university students. J Health Lit. (2018) 3:39–49. 10.22038/JHL.2018.10930

[50] Panahi R Ramezankhani A Tavousi M Niknami S. Adding health literacy to the health belief model: effectiveness of an educational intervention on smoking preventive behaviors among university students. Iranian Red Crescent Med J. (2018) 20:12. 10.5812/ircmj.13773

[51] Liu C Wang D Liu C Jiang J Wang X Chen H. What is the meaning of health literacy? a systematic review and qualitative synthesis. Family Med Comm Health. (2020) 8:351. 10.1136/fmch-2020-000351 32414834 PMC7239702

[52] Mosher HJ Lund BC Kripalani S Kaboli PJ. Association of health literacy with medication knowledge, adherence, and adverse drug events among elderly veterans. J Health Commun. (2012) 17 Suppl 3:241–51. 10.1080/10810730.2012.712611 23030573

[53] Náfrádi L Nakamoto K Csabai M Papp-Zipernovszky O Schulz PJ. An empirical test of the health empowerment model: does patient empowerment moderate the effect of health literacy on health status? Patient Educ Couns. (2018) 101:511–7. 10.1016/j.pec.2017.09.004 28899712

[54] Navarro MD. Patients' empowerment and the role of patients' education. Med Res Arch. (2020) 8:1–7. 10.18103/mra.v8i12.2306

